# Preparation and Characterization of Acrylic and Methacrylic Phospholipid-Mimetic Polymer Hydrogels and Their Applications in Optical Tissue Clearing

**DOI:** 10.3390/polym16020241

**Published:** 2024-01-15

**Authors:** Nanako Dei, Kazuhiko Ishihara, Akikazu Matsumoto, Chie Kojima

**Affiliations:** 1 Department of Applied Chemistry, Graduate School of Engineering, Osaka Metropolitan University, 1-1 Gakuen-cho, Naka-ku, Sakai 599-8531, Osaka, Japanakimatsumoto@omu.ac.jp (A.M.); 2 Division of Materials & Manufacturing Science, Graduate School of Engineering, Osaka University, 2-1 Yamadaoka, Suita 565-0871, Osaka, Japan; k-ishihara@mat.eng.osaka-u.ac.jp

**Keywords:** copolymerization, hydrogel, optical tissue clearing, phosphorylcholine, viscoelasticity

## Abstract

The 2-methacryloyloxyethyl phosphorylcholine (MPC) polymers are mimetic to phospholipids, being widely used as biocompatible polymers. In our previous study, MPC polymer hydrogels proved more effective for optical tissue clearing compared to acrylamide (AAm) polymer hydrogels. In the present study, 2-acryloyloxyethyl phosphorylcholine (APC) was synthesized and employed to create hydrogels for a comparative analysis with methacrylic MPC-based hydrogels. APC, an acrylic monomer, was copolymerized with AAm in a similar reactivity. In contrast, MPC, as a methacrylic monomer, demonstrated higher copolymerization reactivity than AAm, leading to a spontaneously delayed two-step polymerization behavior. This suggests that the polymer sequences and network structures became heterogeneous when both methacrylic and acrylic monomers, as well as crosslinkers, were present in the copolymerization system. The molecular weight of the APC polymers was considerably smaller than that of the MPC polymers due to the formation of mid-chain radicals and subsequent β-scission during polymerization. The swelling ratios in water and strain sweep profiles of hydrogels prepared using acrylic and methacrylic compounds differed from those of hydrogels prepared using only acrylic compounds. This implies that copolymerization reactivity influences the polymer network structures and crosslinking density in addition to the copolymer composition. APC-based hydrogels are effective for the optical clearing of tumor tissues and are applicable to both passive and electrophoretic methods.

## 1. Introduction

Zwitterionic polymers, including sulfobetaine, phosphobetaine, and carboxybetaine incorporate both cationic and anionic groups in the side chain. Due to their unique properties, zwitterionic polymers have been extensively studied for various applications [1,2,3,4,5,6]. The 2-methacryloyloxyethyl phosphorylcholine (MPC) polymer, featuring a phosphobetaine structure, stands out as one of the most popular zwitterionic polymers, mimicking phosphatidylcholine [7,8]. MPC, a methacrylic monomer, readily copolymerizes with other monomers and can be grafted onto a surface. These MPC polymers are useful for surface modification and have been used as materials for medical implants, drug delivery systems, and separation membranes owing to their protein antifouling and low-friction properties [5,8]. MPC hydrogels are prepared by crosslinking MPC polymer chains, forming a three-dimensional network structure with biocompatible properties. These hydrogels are effective in preventing protein adsorption and adhesion, protecting intact cells and tissues, and have found applications as biomaterials in ophthalmic and cell-engineering devices [9,10]. 

Three-dimensional fluorescence imaging has been a focal point for detailed structural analysis to unveil biological significance and clinically relevant features. Various optical tissue-clearing methods have been introduced, including 3D imaging of solvent-cleared organs (3DISCO), clear, unobstructed brain/body imaging cocktails and computational analysis (CUBIC), and clear lipid-exchanged acrylamide-hybridized rigid imaging compatible tissue hydrogel (CLARITY) methods [11,12,13]. In the 3DISCO method, organic solvents are utilized, posing potential harm and damage to proteins. The CUBIC method employs water-soluble reagents, offering a safer alternative but with reduced transparency compared to the 3DISCO method [14,15,16]. In the CLARITY method, proteins are fixed in a polymer hydrogel, and lipid molecules are eliminated through electrophoresis and/or diffusion, followed by a media exchange from water to a liquid with a high refractive index [17,18]. While polyacrylamide (PAAm) hydrogels are commonly used in the CLARITY method, our research has revealed that certain ionic polymers, particularly MPC polymer hydrogels, enhance lipid diffusion from tissues embedded in the hydrogel using the CLARITY method [19,20]. The polymer sequence and composition significantly impact the physicochemical properties of the hydrogels. Monomer reactivity ratios are indices that determine the polymer sequence during copolymerization. In a previous study, we highlighted substantial differences in the monomer reactivities of acrylamide (AAm) and sodium styrenesulfonate (SS). The highly reactive SS formed an SS-rich copolymer in the early stages of radical copolymerization, followed by the homopolymerization of the less reactive AAm after SS consumption. This unique copolymerization property, termed one-shot/two-step radical polymerization, allows the preparation of double-network-like hydrogels using monomers with distinct reactivities in the presence of a crosslinking agent [21,22]. Moreover, acrylic and methacrylic monomers exhibit varying reactivities, despite sharing the same side chain [23,24]. Although there is an abundance of reports on MPC-based biomaterials due to their commercial availability [3,4,5,8,9,10], to our knowledge, there are no reports on three-dimensional network structures containing 2-acryloyloxyethyl phosphorylcholine (APC) where the methacrylic group of MPC is replaced by an acrylic group. 

**Figure 1:**
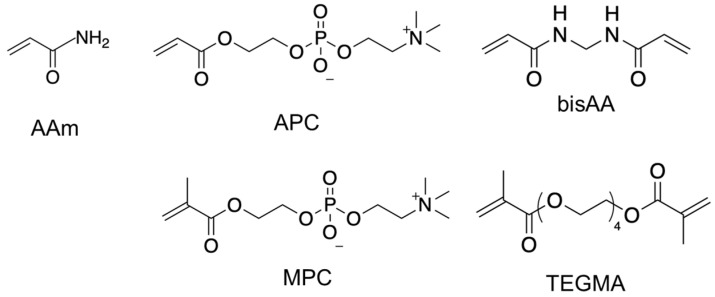
Structures of monomers and crosslinkers used in this study.

In this study, we synthesized and polymerized APC to yield APC homopolymers, copolymers, and their respective hydrogels, providing a basis for comparison with MPC. Initially, the polymerization and copolymerization rates, along with the molecular weights of the resultant polymers, were examined using AAm, APC, and MPC as monomers. Subsequently, hydrogels were formulated utilizing acrylic and methacrylic crosslinkers, as illustrated in Figure 1, enabling an examination of gel fractions, swelling ratios, and viscoelastic properties. Finally, the hydrogels were applied in optical tissue clearing, involving the removal of lipids through both diffusion and electrophoresis. 

## 2. Materials and Methods

### 2.1. Materials

AAm (Nacalai Tesque Inc., Kyoto, Japan), 2-methacryloyloxyethyl phosphorylcholine (MPC) (Tokyo Chemical Industry Co., Ltd., Tokyo, Japan), *N*,*N*’-methylenebis(acrylamide) (bisAA, Tokyo Chemical Industry), and tetraethylene glycol dimethacrylate (TEGMA, Nacalai Tesque) served as monomers and crosslinkers without further purification. The initiator, 2′-azobis [2-(2-imidazolin-2-yl)propane]dihydrochloride (VA-044) (Fujifilm Wako Pure Chemical Industries, Osaka, Japan), was utilized without purification. APC was synthesized following a prior report [25]. Briefly, 2-hydroxyethyl acrylate was reacted with 2-chloro-2-oxo-1,3,2-dioxaphosphorane to produce 2-oxo-1,3,2-dioxaphosphoroyl-oxy-ethyl acrylate, which was then reacted with trimethylamine to yield APC as a white solid. The resulting compound was characterized using ^1^H NMR spectroscopy on an ECX-400 spectrometer (JEOL, Ltd., Tokyo, Japan).

### 2.2. Polymer Synthesis and Characterization

The conversion was assessed through ^1^H NMR spectroscopy, following the methodology shown in our previous report [21]. Briefly, a monomer (AAm, MPC, or APC) was dissolved in D_2_O (0.56 M in total) with 0.14 M NaCl. 2-propanol was added to D_2_O at 10 vol%, which served as an internal standard. Following the addition of the initiator (VA-044, 0.23 wt%, final concentration), the solution was transferred to an NMR tube, and polymerization was performed at 37 °C. Copolymerization was also conducted at a 1:1 molar ratio of AAm to MPC or AAm to APC under the same conditions. After a predetermined time, polymerization was halted with liquid nitrogen, and the ^1^H NMR spectrum was recorded at room temperature. Monomer conversions were calculated based on the change in the integral ratio of characteristic peaks at 5.74 ppm, 5.94 ppm, and 5.67 ppm for AAm, APC, and MPC, respectively, relative to the peak derived from 2-propanol. An induction period, observed in some cases at an earlier stage of polymerization due to the inhibitory effects of oxygen, was excluded.

Following the acquisition of the ^1^H NMR spectra, NMR sample solutions were collected and subjected to an additional 24 h polymerization at 37 °C. The reaction mixture underwent lyophilization and was analyzed by gel permeation chromatography (GPC) to determine the polymer molecular weights. The GPC system comprised TSK gel GMPWXL and TSK gel-α-3000 columns (Tosoh Corp., Ltd., Tokyo, Japan), a PU-2080 Plus pump (Jasco Co., Tokyo, Japan), a CO-2060 column oven (Jasco), and an RI-2031 Plus refractive index detector (Jasco). The polymers were eluted using 0.1 M phosphate buffer (pH 6.8) at a flow rate of 0.5 mL/min and a temperature of 25 °C. The molecular weights were calibrated using standard poly(ethylene oxide)s (*M_n_* = 0.55 k, 2 k, 21.0 k, 44.9 k, 101 k, 185 k, 272 k, 580 k, 895 k, 2000 k, and 4000 k).

### 2.3. Hydrogel Preparation and Characterization

The hydrogels were prepared and characterized as described in our previous reports [19,20]. Briefly, the monomers (0.56 M in total) and crosslinker (bisAA or TEGMA; 6.5, 13, or 19.5 mM) were dissolved in phosphate-buffered saline (PBS). After the addition of VA-044 (0.25 wt%, final concentration), the solutions were incubated at 37 °C for more than 3 h. Subsequently, the obtained hydrogels were immersed in water for 48 h to remove the water-soluble components. Following lyophilization, the dried gels were weighed. The gel fraction and swelling ratio were calculated using the following equations [19,20,26].

Gel fraction (−) = dried gel (g)/(monomer (g) + crosslinker (g))(1)

Swelling ratio (−) = wet gel (g)/dried gel (g)(2)

The wet gel weight was measured after immersing the dried gel in water for 24 h.

The viscoelasticity was assessed using a HAAKE MARS III (Thermo Fisher Scientific, Waltham, MA, USA). The hydrogels used in this experiment were in their as-prepared state without water immersion because some of them dissolved after immersion in water. The columnar hydrogels had a diameter of 20 mm and a height of 2 mm. Strain sweep experiments were conducted over a range of 0.01 to 100% strain at a frequency of 1 Hz, and the storage modulus (G′) and loss modulus (G″) were measured.

### 2.4. Optical Clearing of Tumor Tissues

The optical clearing of tumor tissues obtained from MDA-MB-231 cell-implanted nude mice was conducted as described in our previous reports except for the polymerization time and lipid removal process [19,20]. Briefly, the cut tissues (~8 mm × ~8 mm square and 2 mm thick) were immersed overnight in PBS (pH 7.4) containing monomers (0.56 M in total), a crosslinker (6.5 mM), and VA-044 (0.25 wt%) at 4 °C. Following polymerization at 37 °C for either 3 or 24 h, the hydrogel outside the tissue was removed. Subsequently, the lipids within the tissues were removed through either diffusion or electrophoresis. For the diffusion method, the tissue underwent shaking in 30 mL of 0.8 M borate buffer (pH 8.5) containing 4% SDS at 37 °C for 11 days, as described in our previous reports [19,20]. In the electrophoresis method, the tissues were positioned in a swimming tank (NA-1880, Nihon Eido Co., Ltd., Tokyo, Japan). Electrophoresis was conducted at a constant voltage of 0.5 A (approximately 20 V) using a power hollester (Model-3870; Anatech Co., Tokyo, Japan). Throughout the electrophoresis, 5 L of 0.2 M borate buffer (pH 8.5) containing 4% SDS was circulated using a small chemical-resistant gear pump (GPU-1, As One Co., Osaka, Japan). The buffer temperature was maintained at 37–42 °C using a digital temperature controller featuring a timer function (TC-1NP, As One) and an immersion sheathed heater (stainless steel (SUS316L), 1 kW, LYPDS110, As One). Electrophoresis was conducted during the day (~8 h) for three days, with shaking of the tissue using the diffusion method at night (~16 h). Following lipid removal, any remaining SDS was removed by shaking the tissue in 30 mL of 0.1% Triton X-100 solution for two days. The tissues were subsequently immersed in 20 mL of ethylene glycol for solvent exchange. Images of the tissues, placed on a grid pattern (10 × 10 mm^2^), were captured before and after lipid removal and after the final processing step.

## 3. Results and Discussion

### 3.1. Synthesis and Characterization of Copolymers Containing MPC and APC

**Figure 2:**
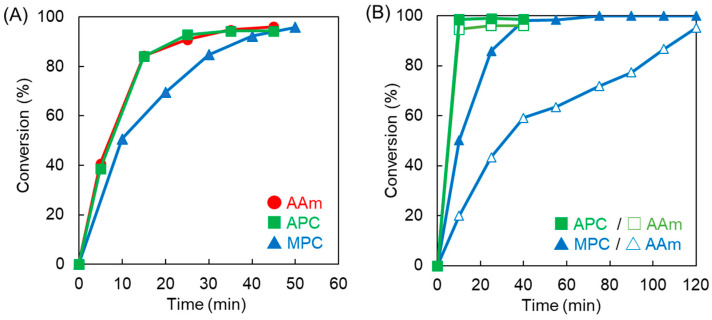
Time-conversion relationship for the (**A**) homopolymerization and (**B**) copolymerization systems of AAm, MPC, and APC.

APC was synthesized following a previously reported method [25], and the obtained monomer was confirmed by ^1^H NMR spectroscopy (Figure S1). Subsequently, the homopolymerization behaviors of AAm, MPC, and APC were examined using ^1^H NMR spectroscopy. The conversion of each monomer was calculated based on the integral ratio of the signal observed at approximately 6 ppm for the vinyl groups to that at 1.2 ppm for isopropanol, which served as an internal standard [21,22]. Figure 2A illustrates the time-conversion relationship for each monomer. APC and AAm were polymerized at the same speed and faster than MPC. This arises from the tertiary radical end of methacrylic MPC in the propagation step being more stable and less reactive than the secondary radical ends of acrylic APC and AAm. Polymerization of these monomers was nearly complete within 1 h. The copolymerization of MPC/AAm and APC/AAm was subsequently conducted at a molar ratio of 1:1. Figure 2B displays the time-conversion relationship for each monomer in the copolymerization system. In the APC/AAm copolymerization, APC and AAm were consumed at the same speed. However, in the MPC/AAm copolymerization, MPC was consumed in advance compared to AAm, resulting in spontaneously delayed two-step polymerization. Previous reports indicate that MPC with a methacrylic group is more reactive than APC and AAm with an acrylic group during radical copolymerization due to the production of tertiary radicals [23,24]. This suggests that the polymer sequence of the APC/AAm copolymer is homogeneous, with all the polymer chains consisting of a 1:1 ratio of APC to AAm. In contrast, MPC-rich random copolymers and AAm homopolymers were likely produced in the early and late stages, respectively, in the MPC/AAm copolymerization. This phenomenon aligns with previous reports on the unique one-shot/two-step radical polymerization performed using monomers with different reactivities [21,22].

### 3.2. Synthesis and Characterization of Polymer Hydrogels Containing MPC and APC

Hydrogels were formulated utilizing MPC, APC, and AAm in the presence of the methacrylic and acrylic crosslinkers, TEGMA and bisAA, respectively (Table 1). Polymerization was conducted for more than 3 h at 37 °C in PBS under varying monomer and crosslinker conditions, followed by immersion in water for purification. The hydrogels were formed under diverse conditions, with the exception of APC and MPC homopolymer hydrogels crosslinked with bisAA and TEGMA, respectively, which were denoted as PAPC and PMPC-t, respectively. These hydrogels readily dispersed in water during purification due to the higher hydrophilicity of APC and TEGMA compared to MPC and bisAA. The molecular weights of the homopolymers and copolymers containing AAm, MPC, and APC without crosslinkers were determined by GPC (Table 2). Both the number-average molecular weight (*M_n_*) and weight-average molecular weight (*M_w_*) of the polymers containing methacrylic MPC were higher than those of the polymers containing acrylic AAm and APC. Additionally, the *M_n_* and *M_w_* of the MPC/AAm copolymer were lower than those of the MPC homopolymer. The peaks of the APC homopolymer and APC/AAm copolymer in the GPC chromatograms were broader than those of the MPC homopolymer and MPC/AAm copolymer (Figure S2). It is known that mid-chain radicals may be formed by hydrogen atom abstraction from the backbone of the propagating chain during the polymerization of acrylate monomers. These mid-chain radicals may induce β-scission, leading to a decrease in the molecular weight of acrylate polymers and hydrogels [27,28]. Thus, it is likely that the polymer network structure of the PAPC hydrogel degraded during polymerization, forming water-soluble micro/nanosized hydrogels.

**Figure 3:**
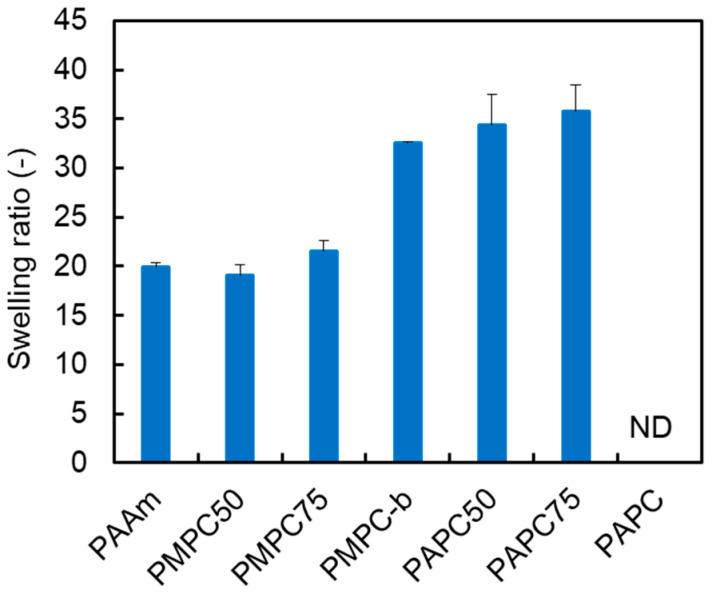
Swelling ratios of various polymer hydrogels in water. The results of the PAAm, PMPC50, PMPC75, and PMPC-b hydrogels were referred to from [19]. ND: not determined.

**Figure 4:**
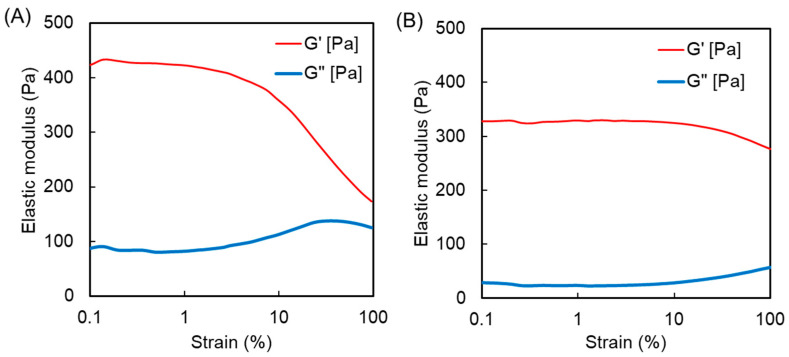
Storage modulus (G′) and loss modulus (G″) of the (**A**) PMPC50 and (**B**) PAPC50 hydrogels versus strain sweep.

We previously reported the swelling ratios of MPC/AAm copolymer hydrogels prepared by crosslinking with bisAA [19]. In the current study, copolymer hydrogels consisting of an APC/AAm copolymer at various compositions were prepared using bisAA as the crosslinker, and their swelling ratios were compared (Figure 3). The MPC homopolymer (PMPC-b) hydrogel exhibited greater swelling than the MPC/AAm copolymer hydrogels. However, the MPC/AAm copolymer hydrogels (PMPC50 and PMPC75) swelled to a level comparable to that of the AAm homopolymer (PAAm) hydrogel, even as the hydrophilic MPC content increased. This observation suggested that the properties of the MPC/AAm copolymer hydrogels were primarily influenced by the AAm–bisAA network structure owing to the differing reactivities of the methacrylic and acrylic monomers/crosslinkers during copolymerization. The swelling ratio of the PAPC50 hydrogel exceeded that of the PAAm hydrogel, indicating that the hydrophilic APC units were incorporated into the polymer network, inducing swelling. However, an increase in the APC content in the APC/AAm copolymer hydrogels did not elevate the swelling ratio, and the PAPC hydrogel remained in a sol state. This was attributed to the degradation of the APC-rich polymer network, forming micro/nanosized hydrogels through β-scission due to mid-chain radical transfer, as discussed earlier [27,28]. To further investigate the polymer network structure in these hydrogels, the elastic moduli, such as the storage modulus (G′) and loss modulus (G″) of the PAPC50 and PMPC50 hydrogels, were measured using a rheometer. The strain sweep profiles of the hydrogels are presented in Figure 4. The storage moduli of the hydrogels were similar in the linear viscoelastic regions. However, the storage modulus of the PMPC50 hydrogel began to decrease at approximately 4% strain, whereas that of the PAPC50 hydrogel remained above 10% strain. This suggests that the network structures of the copolymer hydrogels were different [22]. Polymer networks in most hydrogels are influenced by the crosslinking structure, density, homogeneity, and topological defects, such as dangling chain ends and chain-forming loops [29]. Figure 2 indicates that MPC was predominantly consumed in the early stage rather than AAm and bisAA. This implies that the freestanding and dangling MPC-rich polymer chains were produced at an early stage. The loop structure may have formed from the remaining bisAA at a later stage. Thus, it is possible that the network structure of the PMPC50 hydrogel was more heterogeneous than that of the PAPC50 hydrogel. It was previously reported that the methacrylic group in triethylene glycol acrylate was polymerized in advance compared to the acrylic group [30], which is consistent with our results.

**Figure 5:**
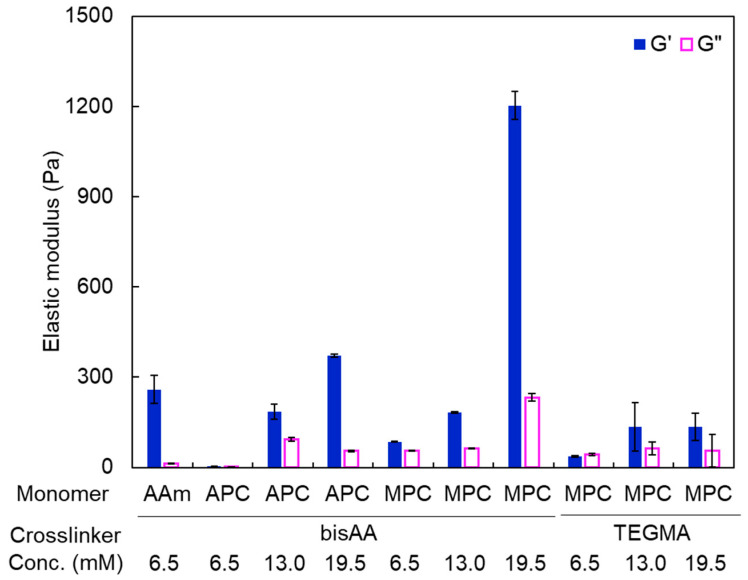
Elastic moduli of the PAAm, PAPC, and PMPC-b and PMPC-t hydrogels prepared at different crosslinker concentrations.

**Figure 6:**
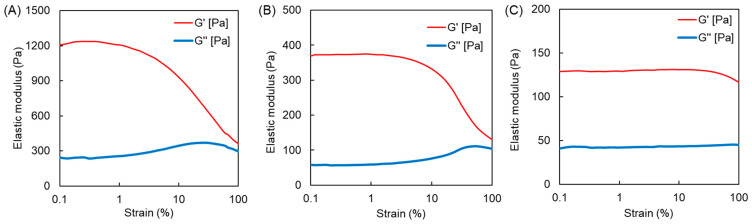
Elastic moduli of the (**A**) PMPC-b, (**B**) PAPC, and (**C**) PMPC-t hydrogels prepared at 19.5 mM crosslinker concentration versus strain sweep.

The APC and MPC homopolymer hydrogels were then prepared using the acrylic and methacrylic crosslinkers, bisAA and TEGMA, at crosslinker concentrations of 6.5 mM, 13 mM, and 19.5 mM, with crosslinker mole percentages of 1.2%, 2.3%, and 3.5% to monomer, respectively. The elastic moduli of the hydrogels were measured (Figure 5). The storage modulus of the PAPC-bisAA (PAPC) hydrogel crosslinked at 6.5 mM of a crosslinker was quite low. The higher loss modulus compared to the storage modulus of the MPC-TEGMA (PMPC-t) hydrogel prepared at 6.5 mM indicates a sol state. However, the MPC-bisAA (PMPC-b) hydrogel had a higher storage modulus than a loss modulus, which is typically observed for gels. These results align with the gel fraction data presented in Table 1, indicating that APC and TEGMA are more hydrophilic than MPC and bisAA, respectively. The storage elastic moduli increased in the PAPC and PMPC-b hydrogels with increasing crosslinker concentration because the hydrogel network became denser. However, the storage elastic moduli did not increase significantly for the PMPC-t hydrogels. The PMPC-t hydrogels became viscous (Figure S3) because the oligoethylene units in the TEGMA were flexible and highly hydrated. Strain sweep measurements of the hydrogels prepared using 19.5 mM crosslinkers were performed (Figure 6). The storage modulus of the PMPC-b hydrogel was the highest (1.1 kPa) and began to decrease at a strain of approximately 1%. The storage moduli of the PAPC and PMPC-t hydrogels were as low as 0.4 kPa and 0.1 kPa, respectively, and were retained to approximately 10% and 50% strain, respectively. This also indicated that the network structures of the hydrogels were different. Although the PMPC-b hydrogel was synthesized using a methacrylic monomer and an acrylic crosslinker, the PAPC and PMPC-t hydrogels were composed of acrylic or methacrylic derivatives. As described above, methacrylic MPC was consumed predominantly at the early stage rather than acrylic compounds, possibly inducing a heterogeneous crosslinking density as well as topological defects. It is likely that the network structure of the PMPC-b hydrogel was more heterogeneous than that of the PAPC and PMPC-t hydrogels. Moreover, the highly flexible tetraoligoethylene linker in TEGMA is a possible cause of the high critical strain of these hydrogels.

### 3.3. Optical Tissue Clearing Using APC- and MPC-Containing Hydrogels

**Figure 7:**
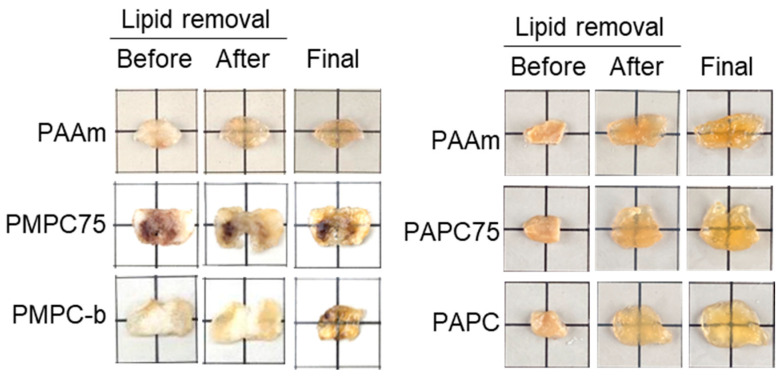
Optical clearing of tumor tissues using PAAm, PMPC75, PMPC-b, PAPC75, and PAPC hydrogels by the passive CLARITY method. The results in the left panel were referred to from [19].

Finally, optical tissue clearing of tumor tissues using APC- and MPC-containing hydrogels was performed to compare the transparency. Optical tissue clearing had been previously conducted using the passive CLARITY method with MPC-containing hydrogels [19]. The tissues cleared using the PMPC-b hydrogels exhibited the greatest transparency after treatment, whereas those treated with the PMPC75 hydrogels exhibited less transparency than the PAAm hydrogel-treated tissues, as shown in Figure 7. The network structure of the PMPC75 copolymer hydrogels was probably heterogeneous owing to the poor copolymerizability of methacrylic MPC and acrylic AAm, as shown in Figure 2, Figure 3 and Figure 4. The APC-containing hydrogels were also used in the CLARITY method to compare the transparency of tissues treated with MPC-containing hydrogels (Figure 7). The transparencies of the tissues cleared using the PAAm, PAPC, and PAPC75 hydrogels depended on the APC composition of the hydrogels The tissues cleared using the PAPC hydrogel exhibited the greatest transparency. The ideal copolymerization reactivity of AAm and APC yields a statistically random copolymer with a corresponding composition, and the swelling ratios and rheological properties are related to the composition. Thus, the polymer composition significantly affects the transparency of the tissue owing to the excellent hydrophilicity of the APC repeating unit. Figure 7 also shows that some tissues expanded after the treatment. Yang et al. reported that expansion does not adversely affect gross tissue morphology or cellular structure [31]. The expansion of the CLARITY method may facilitate microscopic observation because high magnification is not necessary for fluorescence imaging [32]. Thus, expansion does not affect the optical clearing of tumor tissue in the CLARITY method [33,34,35]. Previous reports have also indicated that highly crosslinked hydrogels show lower transparency because the dense network structure inhibits lipid removal from the tissue [32]. Thus, the hydrogels shown in Figure 5 and Figure 6 may be unsuitable for optical tissue clearing.

**Figure 8:**
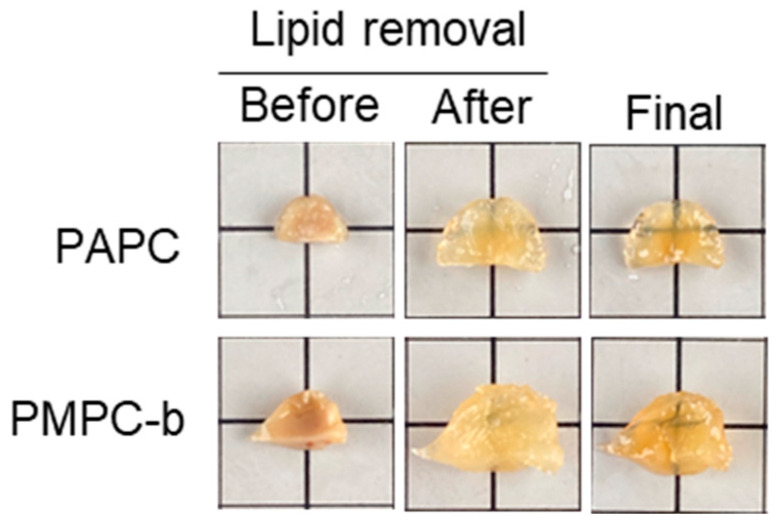
Optical clearing of tumor tissues using PAPC and PMPC-b hydrogels by the electrophoresis-used CLARITY method.

Shortening the processing time of optical tissue-clearing methods holds practical importance. The conventional CLARITY method typically requires one day for polymerizing monomers and crosslinkers [17,18,19,20]. However, as depicted in Figure 2, polymerization was nearly complete within a few hours, reducing the polymerization time to 3 h. Additionally, the lipid removal process in the passive CLARITY method usually demands an extended period. Since diffusion is a primary factor in lipid removal using the passive CLARITY method, it often takes a week or more. To expedite this process, electrophoresis was employed in this study. Nevertheless, tissues embedded in the conventionally used PMPC-t hydrogel suffered significant damage during electrophoresis (Figure S4). The PMPC-t hydrogel, being in a sol state, lacked the strength to support the tissue, as mentioned earlier (Figure 5). In contrast, damage to tissues treated with PAPC and PMPC-b hydrogels was minimal after electrophoresis, with high transparency observed after 3 days in tissues treated with both hydrogels (Figure 8). The PMPC-b hydrogel was in a gel state (Table 2) and maintained its original tissue shape during electrophoresis. Notably, the sol-like PAPC hydrogel exhibited a result similar to that of the PMPC-b hydrogel but different from that of the PMPC-t hydrogel. This might be attributed to the suppression of β-scission by the mid-chain radical in the PAPC hydrogel within the tissue-embedded hydrogel. Since the APC polymer chains formed in the protein networks of the tissues would have a semi-interpenetrating polymer network structure, the tissue embedded in the PAPC hydrogel could retain its entire structure. Our results suggest that APC-based hydrogels are beneficial for optical tissue clearing in both passive and electrophoresis-based CLARITY methods, with effects similar to those of MPC-based hydrogels prepared using a suitable crosslinker.

## 4. Conclusions

In this study, we synthesized an acrylic monomer with a phosphorylcholine group, APC, and assessed its homo and copolymerization reactivity using AAm as a comonomer in comparison with methacrylic MPC. Both APC and AAm are acrylic monomers with similar reactivities during copolymerization, resulting in the formation of statistically random copolymers with a composition corresponding to the monomers in the feed. However, MPC, being a methacrylic monomer, exhibits higher reactivity than AAm as an acrylic monomer, leading to the creation of heterogeneous polymer compositions. Additionally, the chain length of PAPC was shorter than that of PMPC due to the β-scission of mid-chain radicals formed by chain transfer during radical polymerization. The properties of hydrogels prepared using a combination of acrylic and methacrylic monomers/crosslinkers differed from those prepared using acrylic or methacrylic compounds. Our findings suggest that the heterogeneous network structures consist of both acrylic and methacrylic compounds. As demonstrated previously for PMPC hydrogels, PAPC hydrogels are applied to optical tissue clearing based on the CLARITY method. While PMPC-t hydrogel-embedded tissue was destroyed after electrophoresis in the CLARITY method, tissues embedded in PAPC and PMPC-b hydrogels were preserved and exhibited similar transparency in the electrophoresis-based CLARITY method. This study illustrates that the polymer network structure and hydrogel properties depend significantly on the combination of acrylic and methacrylic monomers and crosslinkers. The selection of APC and MPC based on synthesis and usage conditions has been shown to be crucial in designing precise biomimetic polymer materials for biomedical applications.

## Figures and Tables

**Table 1:** Hydrogels in this study.

Name	Monomer (mol%)	Crosslinker	Gel Fraction (-)
PAAm	AAm	bisAA	1 *
PAPC50	APC/AAm (50/50)	bisAA	0.6
PAPC75	APC/AAm (75/25)	bisAA	0.6
PAPC	APC	bisAA	0
PMPC50	MPC/AAm (50/50)	bisAA	0.8 *
PMPC75	MPC/AAm (75/25)	bisAA	0.9 *
PMPC-b	MPC	bisAA	0.9 *
PMPC-t	MPC	TEGMA	0 *

* Referred to from [19].

**Table 2:** Molecular weights of polymers.

Polymer	*M_n_* (/10^5^)	*M_w_* (/10^5^)	*M_w_/M_n_*
PAAm	1.1	4.8	4.4
PAPC	0.13	0.79	6.0
PMPC	2.6	8.4	3.2
PAPC50	0.50	1.7	3.4
PMPC50	1.8	6.4	3.7

## Data Availability

The data supporting the findings of this study are available from the corresponding author upon reasonable request.

## Supplementary Materials

The following supporting information can be downloaded at https://www.mdpi.com/article/10.3390/polym16020241/s1, Figure S1: ^1^H NMR spectrum of APC; Figure S2: GPC chromatograms of synthesized polymers; Figure S3: Photos of the PMPC-t hydrogels crosslinked at (A) 13 mM and (B) 19.5 mM; Figure S4: Optical clearing of tumor tissues using the PMPC-t hydrogel by electrophoresis.
