# Supplementary material for: Preparation and Characterization of Acrylic and Methacrylic Phospholipid-Mimetic Polymer Hydrogels and Their Applications in Optical Tissue Clearing
Source: Polymers (Basel). 2024 Jan 15;16(2):241. doi: 10.3390/polym16020241 (PMC10820725; doi:10.3390/polym16020241)
Supplement: Supplementary file 1 [file polymers-16-00241-s001.zip › polymers-2784264-SI.pdf]

Supporting Information

Preparation and Characterization of Acrylic and Methacrylic Phospholipid-Mimetic Polymer Hydrogels and Their Applications in Optical Tissue Clearing

Nanako Dei[1], Kazuhiko Ishihara[2], Akikazu Matsumoto[1], Chie Kojima[1]*

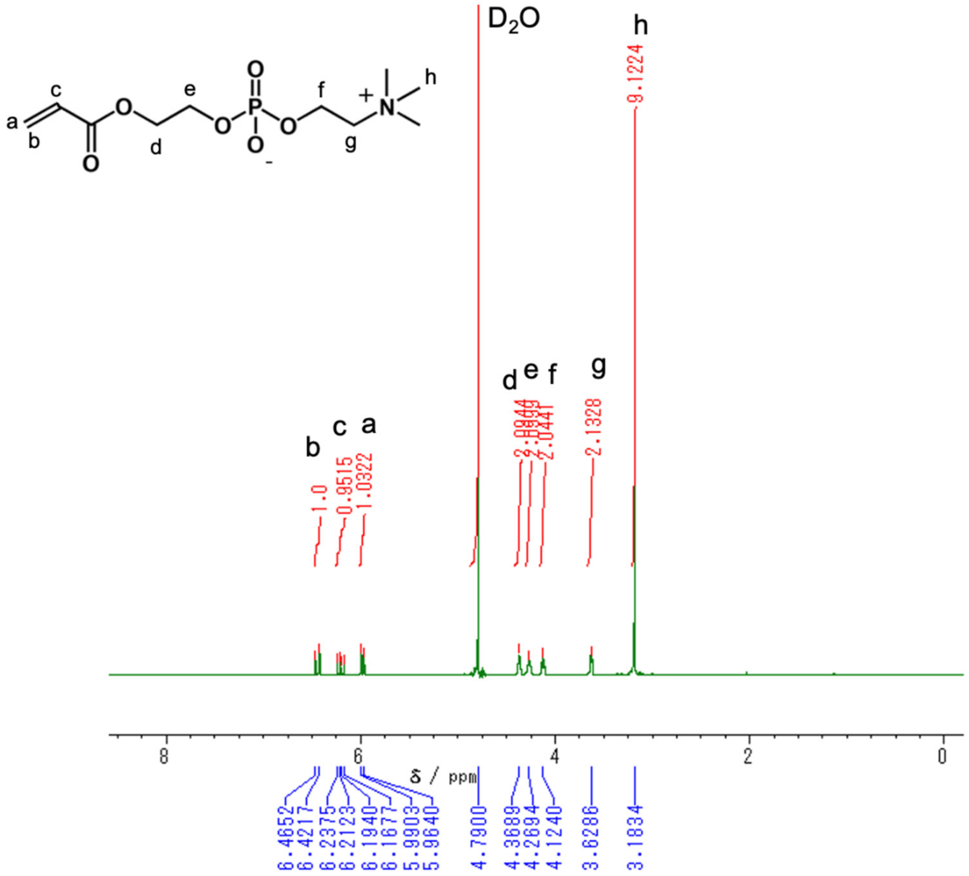

Figure S1. ^{1}H NMR spectrum of APC.

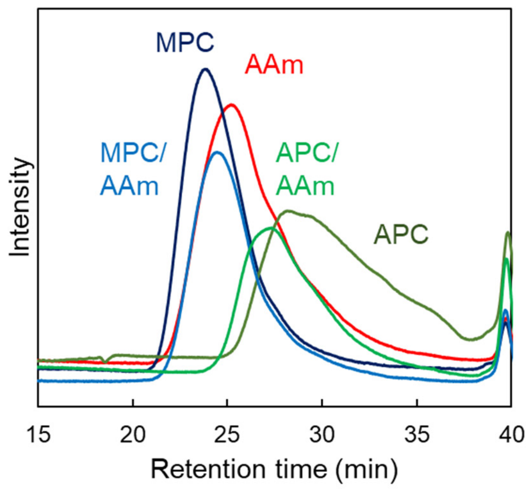

Figure S2. GPC chromatograms of synthesized polymers.

(A) (B)

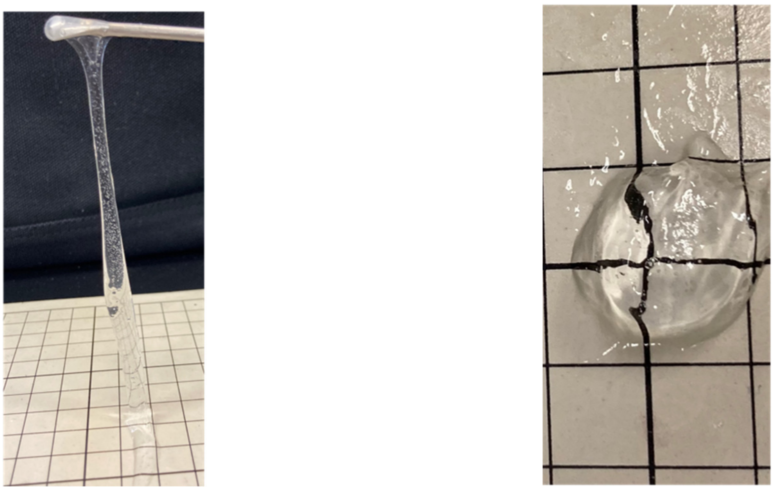

Figure S3. Photos of the PMPC-t hydrogels crosslinked at (A) 13 mM and (B) 19.5 mM.

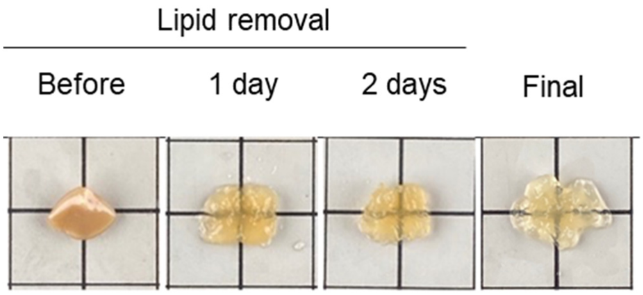

Figure S4. Optical clearing of tumor tissues using the PMPC-t hydrogel by electrophoresis.
